# Left Handedness

**Published:** 1914-03

**Authors:** 


Left Handedness. Bardeleben, *Münch. Med. Woch.*, Dec. 2, 1913, holds that this is not a sign of inferiority. 6.8 per cent. of German recruits are left handed, but about 26 per cent of originally left handed children become right handed by training. About 9 per cent. of human beings are, therefore, originally left handed, and this accounts, by the law of chance, for the small number of left handed persons who have become famous. The gibbon and orangoutang are usually right handed, the gorilla and chimpanzee left handed. Left handedness is really a phase of general muscular left sidedness, with corresponding greater development of opposite nerve centers. It is undoubtedly hereditary in both the familial and racial senses. The real problem is to explain why the majority of persons are right handed.

